# A broad view of pharmaceutical services in multidisciplinary teams of public Primary Healthcare Centers: a mixed methods study in a large city in Brazil

**DOI:** 10.1017/S1463423622000160

**Published:** 2022-05-20

**Authors:** Samara Jamile Mendes, Myllena Farisco, Silvana Nair Leite, Sílvia Storpirtis

**Affiliations:** 1 Pharmaceutical Sciences Department, Faculty of Pharmaceutical Sciences, University of São Paulo, São Paulo, SP, Brazil; 2 Pharmaceutical Sciences Department, Federal University of Santa Catarina, Florianópolis, SC, Brazil

**Keywords:** Brazil, healthcare workers, pharmaceutical services, Primary Health Care, public health systems research

## Abstract

**Aim:**

This study aims to describe how the pharmaceutical services are performed in Primary Healthcare Centers of the Brazilian Public Health System in a large city. *Background:* There is extensive international discussion about the role of pharmacists in health care teams, particularly in Primary Health Care (PHC). However, in Brazil, there is still no consensus on what services the pharmacist should perform in multidisciplinary teams in PHC.

**Methods:**

This study used mixed methods research, and it was conducted with 200 pharmacists who work in PHC Centers of the public health system in São Paulo. The study was conducted using a focus group and an online survey, and qualitative and quantitative data were obtained.

**Findings:**

The analysis of the data from the focus group showed two central themes: (i) pharmaceutical services go beyond medicines and (ii) the contributions of the pharmacist to a multidisciplinary team work in PHC. The survey explored 29 services provided by pharmacists, 7 of which were provided daily. It is important to emphasize that pharmacists do not differentiate the relevance attributed to services considered clinical from those that are managerial or more related to access to medicines. This is an opportunity to develop their teamwork skills. Hence, it is necessary to consolidate the professional identity of the pharmacist and to organize their work processes in a multidisciplinary team. PHC is a space that allows a wide development of pharmaceutical services.

## Introduction

There is extensive discussion around the world on the role of pharmacists in health teams, particularly in Primary Health Care (PHC). Some studies describe the pharmacist as the clinician, the manager, and the social caregiver, a key figure in the community where they work (Pottie *et al*., 2009; Elvey and Hassell, 2013; Hesso *et al*., 2019). Others highlight the transition of a more traditional view of the pharmaceutical services to a more patient-related focus, in which the pharmacist works in a multiprofessional team, investing in training which encourages interprofessional collaborations, different forms of communication, and that defines their role in health care (Schindel *et al*., 2016; Nabhani-Gebara *et al*., 2020).

Recently, studies point to PHC as a fundamental strategy for the development of the health care system (Walley *et al*., 2008). For over 20 years in Brazil, this has been the main strategy used by the Unified Health System (SUS), and it is considered the guiding principle of the SUS (Pinto and Giovanella, 2018).

In 2019, 62.6% of the Brazilian population had access to PHC within the Family Health Strategy (FHS) (Giovanella *et al*., 2021). The SUS offers access to health services, including access to medicines, to all the population; the services are integrally financed by public funds (Castro *et al*., 2019). PHC is based on the performance of a multi-professional team, highlighting the importance of the interdisciplinary nature of the team’s work processes. Multidisciplinary work is the study of an object by different disciplines. It is the sum of insights provided by many areas related to PHC, as well as the different methods from each practice. The challenge of interdisciplinarity is to advance the disciplinary barriers that fragment health care (Alves *et al*., 2004; Luz, 2009).

PHC in Brazil consists of a minimal multidisciplinary team: doctors, nurses, technicians, and a “health agent”, (the community health agent connects the patients/citizen from a determined health district to the professionals of the multidisciplinary team). The other professionals, such as the pharmacist, are part of the teams that support the minimal multidisciplinary teams. Health centers are local establishments distributed in the neighborhoods of all Brazilian cities (Pinto and Giovanella, 2018). Those health centers have an area specifically for the pharmacy, where medicines are dispensed (Leite *et al*., 2017). The Municipal Health Secretariats in the 5,570 districts of Brazil hire pharmacists to work in local public health centers (Faraco *et al*., 2020). The workforce in PHC is mostly constituted by women and 45.5% of these centers have, at least, one pharmacist (Carvalho *et al*., 2017).

In the city of São Paulo, Brazil, there are 363 pharmacists (Sao Paulo, 2018) allocated to 503 PHC Centers of the Public Health System (National Registry of Health Establishments in Brazil, 2018). A study about the insertion of pharmacists in Primary Healthcare Centers in São Paulo showed an important reduction in the shortage of medicines and an improvement in the quality of medical prescriptions, contributing significantly to improve access to medicines and promote their proper use (Melo and Castro, 2017).

However, in Brazil, there is no consensus on the definition of pharmaceutical services (Costa *et al*., 2017) making it difficult for other professionals and patients to understand the role of the pharmacist and their contribution to PHC (Nakamura and Leite, 2016; Silva *et al*., 2018). This study aims to describe the pharmaceutical services performed in Primary Healthcare Centers of the Brazilian Public Health System in a large city. This study used mixed methods research, with data collected from a focus group and an online survey.

## Methods

This study used mixed methods research. They are the collection, analysis, and a combination of both quantitative and qualitative techniques in the same research design. The interaction between them offers better analytical possibilities (Creswell and Plano Clark, 2011). The study was conducted between November 2016 and June 2017, based on data collected from a focus group and an online survey. The focus group results contributed to the creation of the survey. The information from the qualitative stage was used in the quantitative one.

### Sample description

All pharmacists who participated in this study work in Primary Healthcare Centers (PHC) of the Public Health System in São Paulo. Pharmacists work in different PHC units, some with outpatient facilities and spontaneous demand and others with the Family Health Strategy, which is the Brazilian PHC model (Castro *et al*., 2019). All study participants were recruited based on an agreement between the University of São Paulo and the Municipal Health Secretariat of São Paulo/SP (MHS-SP). In both stages (a focus group and an online survey), an invitation email was sent to the contact list provided by MHS-SP.

In Brazil, there are 501 pharmacy undergraduate schools distributed among 2,864 higher education institutions (in 2016). The pharmacy schools in Brazil offer a Bachelor’s Degree Program which allows students to perform academic or professional activities in the pharmacy field (Lopes *et al*., 2019). Many pharmacists take postgraduate courses in clinical pharmacy or continuing education courses focused on public health (Manzini *et al*., 2021).

### Data collection

For the qualitative data collection, a focus group is an instrument based on the participants’ ability to form opinions and attitudes based on the interaction with other individuals (Pope *et al*., 2000). For the focus group, there were 20 participants from the Municipal Health Secretariat of São Paulo/SP. The fulfillment of these 20 vacancies on this study was made by randomly choosing names from the MHS-SP pool list. The activity was coordinated by one of the researchers, and there was also an external observer. A script was used with the following topics: 1) the group’s understanding of pharmaceutical service; 2) pharmaceutical services for medicines and people; and 3) the social importance of the services developed by the participants. The discussion was recorded and later transcribed by the research team. The focus group lasted two hours. The focus group participants were also asked to describe, by using cards, the services they develop in their health unit.

The survey was based on the focus group results and on the literature about clinical pharmacy and medicines management (Manzini and Mendes, 2015; Melo and Castro, 2017). The collection of quantitative data happened with the use of the online form (Google® Form), with a list of 29 pharmaceutical services.

For each service, there were four questions: (1) whether the pharmacist is the one who performs the service; (2) how often the service is provided (daily, weekly, fortnightly, monthly or yearly); (3) the degree of importance of services in the context of the work process; and (4) the degree of importance of services based on the pharmacist’s expectations of an ideal work process. For the answers to the last two categories, the Likert scale from 1 to 5 was used (1– unimportant and 5 – very important).

The survey was sent to 162 pharmacists, representing 40% of São Paulo pharmacists, who had voluntarily registered to participate in researches at the University of São Paulo.

### Data analysis

The focus group analysis was carried out through themes analysis (Pope *et al*., 2000). After extensive reading of the focus group transcripts, it was possible to make some inferences from the meaning of what was said. In the search for key points from the interpretation of the quotations, two main themes emerged. In order to clarify these results, excerpts from the transcripts of the participants’ speeches are presented.

Data from the online form were analyzed using descriptive statistics in Microsoft Excel 2010®. The method was also validated by the COREQ checklist, referring to the focus group and online form (Tong *et al*., 2007).

### Ethics

This study was approved by the Research Ethics Committee of the Faculty of Pharmaceutical Sciences of USP and the Municipal Health Secretariat of São Paulo/SP. The participants provided written informed consent.

## Results and discussion

### Results obtained from the focus group

Twenty (20) pharmacists participated in this stage of the study. The average age was 35 years old, 45% of participants graduated between 5 and 10 years ago and 25% less than 5 years ago. Concerning the time working in the SUS, 35% have worked in the SUS for a period between 5 and 10 years and 50% for less than 5 years. Therefore, the sample is heterogeneous, as there is a mixture of new and more experienced pharmacists.

After analyzing the focus group data, two central themes emerged.

### Pharmaceutical services go beyond the medicine

- *“Do we pay attention to health promotion? Providing the patient with medication that will cure or treat him is useless if they have no guidance, we are there between the medicines and the patient as we provide them this information.” (SIC)*
- *“Both in inventory management and guidance on how to use medications properly, or how to get the medication through the SUS, we promote access, regardless of the procedure stage.” (SIC)*
- *“The access to health, medications, and treatment, to name a few, is promoting health. The health promotion she [another participant] just mentioned here, from the orientation, you can control the inventory of this medication so you are able to provide the medication for that patient, and thereby to promote health.” (SIC)* From these direct quotations, the pharmacists demonstrate the breadth of their services and the possibilities of reorienting their work towards people’s care, being the professionals responsible for ensuring access to and adequate use of medicines.

In 2006, the WHO already defined pharmaceutical services as a set of actions in the health system that aim to ensure comprehensive and continuous attention to the population’s health needs, both individual and collective, with medicines as one of the elements to be employed (WHO, 2006). In Brazil, the change in focus of the pharmacist’s work processes from medicines-centered to people’s health care has a direct relationship with the development of the SUS.

The participants understood that the pharmacist could reconstruct the notion of health care as restricted to the use of medicines, which is closest to the PHC guidelines:

- *“the interesting part for them is to obtain the medication, the patient is very focused on the medicines and we, as pharmacists, have to take the focus off the medication.” (SIC)*
- *“When you question the services, the services should be way beyond just the medicine, in my view, you know? The moment the patient has any complaints, any situation that goes beyond the medication, I think we as health professionals can contribute.” (SIC)*

Stimulated by the society that consume them, medicines became a mixture of consumer goods and therapeutic instruments, and these factors were decisive for the pharmacist to take distance from individual care (Angonesi and Sevalho, 2010; Pereira and Freitas, 2008).

Taking advance of this scenario, participants have an expanded understanding of their services, such as guidance to help people, from guidelines on how to access medicines to therapeutic monitoring, or just inventory management, as illustrated in the transcripts:

- *“From promoting access to this orientation to an effective follow up and also the inventory management part, because one thing leads to another.” (SIC)*

When asked about their services goals, pharmacists were able to state that their services are performed with a focus on people rather than on medicines and made the following statements:

- *“for people, but patients are very attached to medicine.” “For both.” (SIC)*

The resignification of pharmaceutical services in this area can be described by the concept of social constructionism, which investigates the way social phenomena are produced and challenge conventional ideas, demystifying the *status quo* (current state of affairs) of phenomena as it comprises, they are created through historical processes and social interactions (Giddens and Sutton, 2016). Thus, pharmaceutical services built in conjunction with other services provided in PHC challenge the normativity that a pharmacist is not a health professional who is able to take care of people.

### The contributions of the pharmacist to a multidisciplinary team work in PHC

- *“We can take care of the patient dealing not only with medication but also with any other problem that may happen, rather than just being there. I think [a pharmacy] has a broader and more strategic view than most health services.” (SIC)*

Many activities could be developed by the pharmacist in the multidisciplinary teams in PHC, which is an area of important investment by health systems.

Internationally, there is a discussion related to the advances of the participation of pharmacists in PHC teams (Dolovich *et al*., 2008), as well as the health results for chronic diseases with a better use of medicines, which are possible due to pharmacist interventions (Tan *et al*., 2014).

Some participants pointed out that, despite new possibilities, there are still barriers for services to be performed apart from the focus on the medicines. The PHC model provides other options, such as the encouragement of therapeutic groups, home visits, and working with the team:

- *“So, I try to show them [patients] what alternatives they have that may decrease the amount of medicines taken at that moment, right? Because they don´t usually really know.” (SIC)*
- *“Together with the doctor or nurse in the discussion of clinical cases, we are there working with the whole team, in a case discussion, pharmacotherapy, and everything else.”* (SIC)The role of the pharmacist in the multidisciplinary team can be influenced by external and internal factors. There is a need for pharmacists with proactive attitudes and who demonstrate how they can act for the patient’s care, as shown below:
- *“Because if we don’t understand it, if we don’t have a positive attitude, and demonstrate that we can contribute to the health care, to the patient, nobody will go to a pharmacy, they just think that the pharmacy is for taking their medicines and leave… many teams also think that.” (SIC)*

It is important for the pharmacist to develop skills that are applied in the context of teamwork, such as being responsible or co-responsible for the pharmaceutical service, being able to directly perform a procedure or develop a practice, supervise and monitor the performance by another worker, who is properly trained and qualified, or to perform an action together with another professional or health team (Campese, 2017).

Finally, when discussing the importance of their services, they say that although they encounter barriers, they know how important their jobs are because, in PHC, the bonding and trust are legitimized when there are home visits, for example, and they are linked to real-life contexts:

- *“At home visits, you can see the patients’ home, where they store [their medicine]. Also, because it is difficult for the patient to return to the pharmacy [after the home visit], I [pharmacist] go to their place.” (SIC)*

Some studies have already demonstrated advances in the pharmacist work in PHC. However, there are still challenges related to the incorporation of new activities into their work processes and the consolidation of their identity in the multidisciplinary team (Silva *et al*., 2018).

Between 2008 and 2013, the number of pharmacists registered in PHC Centers in Brazil grew 75.0%. Two factors possibly enabled it: the implementation of the Family Health Support Center and the growth of pharmaceutical education and services in the country (Carvalho *et al*., 2017).

In Brazil, supporting the Family Health teams there is the Family Health Support Center (NASF, in Portuguese), which expands the scope of Primary Health Care. The insertion of pharmacists in this multidisciplinary context represents an opportunity to improve the work process and the access to medicines and their rational use (Nakamura and Leite, 2016).

Furthermore, Brazil is one of the few countries that has a public pharmaceutical service model in which pharmacists coordinate all activities related to the medicine chain in government spheres, from selection to use (Carvalho *et al*., 2017).

### We have a lot of work to do: Results of the online survey

A total of 134 responses (83% of the sample) were obtained, 18 of which were excluded (3 duplicates, 7 pharmacists who had participated in the focus group, and 8 unidentified). In the end, 116 responses to the form were analyzed.

Of the pharmacists included, 83.5% were female, all older than the age of 25, and 40% graduated more than 10 years ago. It is noteworthy that 46.5% of the pharmacists were hired by the Public Health System in São Paulo from 01 to 05 years ago, and the majority (92.2%) worked between 31 and 40 h weekly. Regarding their experience working at the SUS, 47% were hired between 5 and 10 years ago.

Pharmaceutical services performed in Primary Health Care are shown in Table 1. Between 70% and 90% of the pharmacists indicate that they perform 12 services in PHC. Eleven types of services are performed by 60% to 30% of pharmacists and only 3 services are performed by less than 5% of them.

**Table 1.** Pharmaceutical services done in PHC of São Paulo, Brazil, based on the perception of the study participants

Pharmaceutical services	Realization (%)
Medicine stock control	99%
Patient guidance to access to and use of medicines	97%
Psychotropic medicine control	97%
Dispensing of medicines and other materials	97%
Pharmacy team supervision	97%
Pharmacy team training	97%
Inform other health care team about medications	96%
Manage medicine storage conditions	96%
Stock control of other materials and health supplies	78%
Documentary record of the services provided	78%
Therapeutic Support Groups	75%
Disease prevention and health promotion actions	72%
Request for medicines extra the list standardized by the municipality	67%
Revision of prescriptions	63%
Discussion of clinical cases with the health team	63%
Pharmacotherapeutic follow-up	60%
Home visit	59%
Pharmacist’s participation in health unit planning actions	57%
Participation in family health team meetings	51%
Guidance from community health workers	47%
Medication reconciliation	35%
Participate in the therapeutic choice of patients with the health team	28%
Evaluate signs and symptoms	28%
Medicine acquisition	9%
Medicine selection	7%
Participation in the Municipal Health Council	5%
Prescribing over-the-counter medications	2%
Participation of the Pharmacy and Therapeutics Commission	2%
Vaccination Services	0%

Brazilian pharmacists have developed an expertise to check the availability of medicines in the SUS: the access routes, inventory management to meet local needs, meetings with the health team to discuss the availability of medicines, and what is prescribed. All services are still under development.

Other studies show similar situations. There is a large number of pharmacists in community pharmacies, and their workforce has increased in recent years. However, the services they develop still need to be better used (Campbell *et al*., 2017). In Canada, for example, pharmacists perform more than 20 PHC services. All the pharmacists reported, in an exploratory study, that they are engaged in direct patient care, including therapeutic issues, drug overhauls, and post-hospitalization drug reconciliation (Gillespie *et al*., 2017).

Another aspect considered for each pharmaceutical service was the frequency of performance. Table 2 shows the frequency of services that 90% of pharmacists claim to perform.

**Table 2.** Pharmaceutical services in PHC of São Paulo, Brazil, reported as the most frequent ones

Pharmaceutical services	Frequency (%)
Pharmacy team supervision	Daily (91%)
Dispensing of medicines and other materials	Daily (87%)
Patient guidance to access to and use of medicines	Daily (87%)
Psychotropic medicine control	Daily (86%)
Manage medicine storage conditions	Daily (62%)
Medicine stock control	Daily (61%)
Inform other health care team about medications	Daily (44%)
Pharmacy team training	Monthly (39%)

Of all services performed daily, only one has direct interaction with the multidisciplinary team: informing other health care teams about medicine. The other services (Table 2) of the pharmacist’s work routine are specific to the pharmacy.

The work with the pharmacy team (which consists of pharmacy technicians) stands out. In Brazil, the technicians are a fundamental part of the health workforce. The pharmacy workforce in dispensing service at a PHC is made up of 43% of technicians and 33.3% of pharmacists (Carvalho *et al*., 2017).

For each service, the pharmacists indicated that the degree of importance and the average answers (1 – unimportant and 5 – very important) are presented in Table 3. It is possible to observe that pharmacists understand their services as important, not differentiating the types of services as more or less important. This is essential, as pharmacists value their work in PHC and recognize themselves as a crucial part of this process.

**Table 3.** Degree of importance of the pharmaceutical services in the current context and ideal work process in the PHC, according to the pharmacists’ perception

Pharmaceutical Services	Degree of importance of the service in the current context of the work process	Degree of importance of the service according to the pharmacist’s expectation of an ideal work process
	Average (SD)	Average (SD)
***Services that pharmacists expect most importantly***		
Patient guidance to access to and use of medicines	4.76 (±0.96)	4.89 (±0.45)
Psychotropic medicine control	4.75 (±0.71)	4.85 (±0.53)
Dispensing of medicines and other materials	4.55 (±0.87)	4.68 (±0.67)
Pharmacy team supervision	4.55 (±0.86)	4.89 (±0.43)
Pharmacy team training	4.48 (±094)	4.90 (±0.45)
Medicine stock control	4.48 (±0.89)	4.73 (±0.57)
Manage medicine storage conditions	4.34 (±0.96)	4.46 (±0.89)
Therapeutic Support Groups	4.33 (±0.93)	4.77 (±0.58)
Documentary record of the services provided	4.32 (±1.07)	4.77 (±0.62)
Inform other health care team about medications	4.29 (±1.08)	4.86 (±0.48)
Disease prevention and health promotion actions	4.25 (±0.97)	4.85 (±0.50)
Revision of prescriptions	4.03 (±1.33)	4.90 (±0.44
Discussion of clinical cases with the health team	4.01 (±1.29)	4.81 (±0.49)
Home visit	4.00 (±1.22)	4.69 (±0.66)
Pharmacist’s participation in health unit planning actions	3.88 (±1.33)	4.70 (±0.64)
Guidance from community health workers	3.85 (±1.42)	4.61 (±0.95)
Medicine selection	3.81 (±1.42)	4.59 (±0.80)
Participation in family health team meetings	3.80 (±1.42)	4.58 (±0.94)
Pharmacotherapeutic follow-up	3.80 (±1.40)	4.81 (±0.54)
Participation of the Pharmacy and Therapeutics Commission	3.79 (±1.46)	4.52 (±0.91)
Evaluate signs and symptoms	3.77 (±1.26)	4.42 (±0.85)
Participate in the therapeutic choice of patients with the health team	3.75 (±1.43)	4.76 (±0.54
Medicine acquisition	3.72 (±1.34)	4.11 (±1.16)
Medication reconciliation	3.69 (±1.40)	4.57 (±0.85)
Participation in the Municipal Health Council	3.69 (±1.26)	4.21 (±0.93)
Vaccination Services	3.43 (±1.49)	3.76 (±1.33)
Prescribing over-the-counter medications	2.95 (±1.44)	3.67 (±1.34)
***Services that pharmacists expect to be less important***		
Request for medicines extra the list standardized by the municipality	4.89 (±1.19)	4.23 (±0.99)
Stock control of other materials and health supplies	4.04 (±1.25)	3.94 (±1.30)

The pharmaceutical services models in Brazil, not contextualized using the PHC principles, have proved to be fragmented, with divisions of service groups considered clinical x managerial ones, and the ones called middle activities for care and clinical services (Correr *et al*., 2011; Pereira *et al*., 2015). In 2000, Storpirtis *et al*. stated that pharmacists should act in an integrated manner, which would certainly benefit the population and could reduce health spending (Storpirtis *et al*., 2000).

The right of access to medicines is ensured by ubiquitous public healthcare centers (Castro *et al*., 2019). Pharmacists are playing an increasing role in PHC centers, fulfilling a growing range of roles and responsibilities, especially for improving access to medicines and their appropriate use (Faraco *et al*., 2020).

PHC has guidelines that provide opportunities for the pharmacist to act in health care such as assistance during the first contact with the SUS, care coordination, longitudinal care plans, mechanisms to ensure accessibility, as well as patient embracement (Portela, 2017).

The study’s limitations were the difficulty of contacting pharmacists at the Centers of the Unified Health System in São Paulo. Professionals find it difficult to participate in research and activities that force them to leave the center, as there are many responsibilities and patients to take care of. More than one focus group could have been created, as there were enough participants. However, at that time, the decision was to keep a larger number of participants in the group, as evasion was expected, which did not happen.

## Conclusion

This study seeks to answer the question of what role a pharmacist has in PHC. According to the perception of PHC pharmacists, it was possible to identify two central themes, which are pharmaceutical services go beyond the medicine and the contributions of the pharmacist to a multidisciplinary teamwork in PHC.

It was also possible to catalogue 29 services provided by pharmacists. The list of services shown has not been divided into service groups. This is because there is an understanding that services are not fragmented. It is important to emphasize that pharmacists do not differentiate the relevance attributed to services considered clinical from those that are managerial or more related to access to medicines. This is an opportunity for them to develop their teamwork skills. Hence, it is necessary to consolidate the pharmacist’s professional identity and organize their work processes in a multidisciplinary team. PHC is a space that allows a wide development of pharmaceutical services.

More research is needed in the area, such as the investigation of the relationship between pharmacist training in Brazil and the development of skills and competences to work in health care and in PHC. In addition, studies that use qualitative methods can help further deepen the role of the pharmacist in PHC’s multidisciplinary teams.
